# Racial segregation and mental health service use by older adults

**DOI:** 10.1093/geroni/igab046.3429

**Published:** 2021-12-17

**Authors:** Kyeongmo Kim, Denise Burnette

**Affiliations:** 1 Virginia Commonwealth University, Henrico, Virginia, United States; 2 Virginia Commonwealth University, Virginia Commonwealth University, Virginia, United States

## Abstract

Older adults living in racially segregated neighborhoods often lack access to mental health care. This study assessed the role of racial segregation in mental health service use and examined whether the relationship between segregation and mental health service use differs by race/ethnicity. We linked residential segregation data from the National Neighborhood Change Database to the 2015 Medical Expenditure Panel Survey. The sample included 4,023 adults aged 65 and older. We measured mental health service use as visit(s) to a mental health professional and/or use of prescribed medication for mental health (1=yes, 0=no) during the past year. Residential segregation was assessed using a combined measure of isolation (level of interaction with the same racial and ethnic group members) and dissimilarity (evenness of distribution of racial groups). Indices ranged from 0 (integrated) to 1 (segregated). We adjusted for age, sex, race/ethnicity, marital status, education, income, attitude toward health care, health insurance, and mental health status. Multiple logistic regression analyses showed that older adults living in more segregated counties were less likely to use a mental health service than those living in more integrated counties (OR=0.77, p=.04). The relationship did not differ by race/ethnicity. As expected, Blacks and Hispanics underused mental health services compared to Whites. The findings highlight that racial segregation limits access to mental health care. Practitioners and policy-makers should identify mental health needs and service use patterns to target services effectively and efficiently. Future research should explore the intersection of income and mental health care resources in segregated neighborhoods.

